# The double-edged nature of multidimensional perfectionism: career adaptability in employment stress among college students

**DOI:** 10.3389/fpsyg.2026.1845501

**Published:** 2026-08-31

**Authors:** Jianhua Liu, Zengjun Yang

**Affiliations:** 1 School of Economics and Management, Yunnan Technology and Business University, Kunming, Yunnan, China; 2 School of Business, Lanzhou University of Information Science and Technology, Lanzhou, Gansu, China

**Keywords:** career adaptability, college students, employment stress, negative perfectionism, positive perfectionism

## Abstract

**Background:**

As job opportunities become more competitive, the job search process has become a salient source of stress for college students. From a psychological perspective, differences in stress experiences are closely related to how individuals manage and expend their psychological resources.

**Methods:**

Based on Conservation of Resources theory, this study used well established scales and conducted a survey among 900 college students from 3 universities in China. The study focused on perfectionism as an individual difference characteristic, examined the associations between its different orientations and employment stress among college students, and further explored the role of career adaptability in these associations.

**Results:**

The results showed different association patterns for the two forms of perfectionism. Positive perfectionism (organization- and orderliness-oriented perfectionism) was associated with lower levels of employment stress, whereas negative perfectionism was more often accompanied by higher levels of employment stress. In addition, career adaptability showed indirect associations between both forms of perfectionism and employment stress.

**Conclusion:**

These findings provide empirical evidence for understanding differences in psychological resources among college students during the job search stage and offer references for the development of related support initiatives.

## 1 Introduction

Against the background of an increasingly complex employment environment and continuously intensifying job competition, college students generally face notable employment stress in the process of moving from campus to the workplace (Peng et al., 2024; Tu and Liu, 2025). Employment stress is usually defined as the psychological tension experienced by individuals during job seeking and career transition when they perceive a gap between external demands and their own resources and abilities (Tu and Liu, 2025). Continued employment stress is associated with harm to emotional stability and mental health of college students, and it is also associated with negative outcomes in job search efficiency, career decision making, and employment confidence (Wu et al., 2024). However, even in similar employment situations, different students still showed some differences in their perceived stress and adaptation outcomes (Peng et al., 2024; Wang et al., 2023). This suggests that employment stress is not only a direct result of changes in the external employment environment, but it is also associated with how individuals view job demands and how they evaluate their own coping ability. Based on this, identifying key personality traits and psychological resources that are associated with college students’ employment stress experience may be an important entry point for understanding the formation process of employment stress. Perfectionism is defined as a stable personality tendency in which individuals pursue high standards and flawlessness. It is not only reflected in the pursuit of excellence and order, but also in concerns about mistakes, strict self-evaluation of performance, and sensitivity to external evaluation (Frost et al., 1990; Simon et al., 2025). This trait may be particularly prominent when facing job-seeking situations with high uncertainty, strict evaluation criteria, and significant outcomes (Zhang et al., 2025). At the same time, career adaptability is associated with the psychological resources and abilities that individuals use to deal with tasks, transitions, and uncertainty in the process of career development (Ouyang et al., 2025). Therefore, examining how perfectionism and career adaptability are related to employment stress may help to understand the potential mechanism behind differences in students’ psychological experiences in job seeking situations.

Previous studies have shown that perfectionism is significantly associated with career adaptability, and perfectionism is also related to employment stress among college students (Chen et al., 2022; Marcionetti et al., 2025). However, most existing studies mainly focus on separate examinations of pairwise relationships between variables. They have not systematically examined, within one framework, how perfectionism is associated with employment stress through career adaptability as a psychological resource. At the same time, although prior research suggests that different perfectionism tendencies may be related to different psychological adjustment outcomes in college students, this issue still lacks systematic testing in the context of job transition. Based on this, the contribution of this study is not only to include employment stress as an important outcome variable, but also to integrate perfectionism, career adaptability, and employment stress in one model, and to test both the direct associations between different dimensions of perfectionism and employment stress and the indirect associations through career adaptability. Through this integrated analysis, this study may help to further clarify the different roles of perfectionism in the job transition stage of college students. It may also provide support for related research on perfectionism, career development, and stress adjustment, and it may also provide a basis for universities to develop more targeted psychological interventions for employment and career counseling.

## 2 Theory and hypotheses

### 2.1 Theoretical foundation

Conservation of Resources theory (COR) suggests that individuals have a basic motivation to protect their existing resources, maintain the stability of resources, and obtain new resources. Stress is more likely to occur when resources are threatened, lost, or when existing resources are insufficient to meet situational demands (Hobfoll, 1989). For college students in the job transition stage, the employment context is often accompanied by competition, selection, and uncertain feedback. This not only requires individuals to continuously invest time, energy, emotional, and cognitive resources, but it also means that their existing resources are more likely to face depletion and threat (Wu et al., 2024). Therefore, employment stress can be understood as a situational psychological response that occurs when individuals perceive a lack of resources, a threat to resources, or an increase in resource loss during the job search process.

In the field of perfectionism research, the ways of classifying perfectionism are diverse (Grugan et al., 2021; Kahn et al., 2023). This study mainly follows the classic multidimensional perspective of Frost et al. (1990). Based on its dimensions, perfectionism will be classified into positive perfectionism and negative perfectionism (Chen et al., 2022; Tang et al., 2026). This is used to examine the different patterns of these two types of perfectionism in the employment context. Positive perfectionism usually makes individuals more likely to organize their efforts around set goals and to invest resources in task preparation and continuous action. In contrast, negative perfectionism is more likely to make individuals focus on mistakes, failures, and negative evaluations. This is associated with higher self-monitoring, more worry, and greater emotional resource consumption (Simon et al., 2025). According to COR, this difference suggests that different dimensions of perfectionism may be associated with different levels of resource conservation or resource loss risk during the job search process (Maftei and Opariuc-Dan, 2023), and may also be associated with different levels of employment stress. However, focusing only on perfectionism as a relatively stable personality trait is still not enough to fully explain differences in stress among college students in specific job seeking situations. According to COR, stress is closer to a subjective judgment of whether current resources are sufficient to deal with external demands (Hobfoll, 1989). Perfectionism is more located at the early stage of the resource process. It reflects a stable tendency of individuals in how they allocate and use resources in long term goal pursuit (Schmitt et al., 2021). Therefore, between perfectionism and employment stress, it is still necessary to include a variable that reflects the current state of resource readiness. Within this theoretical framework, career adaptability can be understood as a psychological resource reserve that individuals can draw on in career transition situations (Savickas, 1997; Wang et al., 2021). It is closer to the actual state of resources than perfectionism, and it is also closer to the direct process of employment stress formation than stable personality traits (Ouyang et al., 2025). At the same time, prior research has shown that career adaptability is significantly associated with perfectionism tendencies among college students (Chen et al., 2022), and it is also closely related to adaptation experiences and stress levels in the job search stage (Ling et al., 2024). Therefore, from the perspective of COR, positive perfectionism and negative perfectionism can be viewed as personality factors that are associated with how individuals maintain and lose resources. Career adaptability can be viewed as a resource readiness state related to career transition. Employment stress reflects a situational experience based on the match between resource demands and resource conditions. Based on this theoretical logic, this study includes positive perfectionism, negative perfectionism, career adaptability, and employment stress in one analytical framework. It aims to examine the psychological adaptation process of college students in the job transition stage, from resource maintenance and loss to resource readiness state, and then to stress experience.

### 2.2 Hypotheses

#### 2.2.1 Perfectionism and employment stress

Perfectionism is regarded as a personality trait composed of multiple psychological components (Frost et al., 1990). In this study, based on research in Chinese samples by Tang et al. (2026) and Chen et al. (2022), perfectionism is divided into positive perfectionism and negative perfectionism. Positive perfectionism in this study specifically refers to the organization and orderliness-oriented dimension, reflected in individuals organized and structured approaches to task completion and relatively positive self-management behaviors. Negative perfectionism primarily reflects stronger concern over mistakes, self-doubt, high personal standards, sensitivity to external expectations, and greater psychological burden when individuals face demands and evaluations. Although in the existing literature, adaptive perfectionism typically comprises two core components, personal standards and organization, subsequent factor analytic studies have shown that the factor loading of personal standards is unstable across different samples and is positively correlated with psychological distress such as anxiety and depression (Koutra et al., 2023; Simon et al., 2025). Based on these considerations, personal standards are classified under the negative perfectionism dimension in this study.

Studies that directly examine the relationship between organization and orderliness-oriented perfectionism and employment stress are relatively limited. However, Simon et al. (2025) found that adaptive perfectionism is generally accompanied by more positive emotional experiences and higher levels of self-evaluation. This corresponds to some extent with the positive perfectionism defined in this study in terms of conceptual function. In contrast, Koutra et al. (2023) note that negative perfectionism involves excessive concern over mistakes, self-criticism, and fear of failure, and it more frequently co-occurs with anxiety, tension, and elevated stress experiences. Together, these findings suggest that different forms of perfectionism may show distinct patterns in college students’ stress perceptions and emotional responses. Within employment contexts, college students often face multiple and concurrent demands, and these contextual features may further intensify associations between internal traits and external pressures. Kwak et al. (2025) and Powell et al. (2021) describe employment contexts as highly competitive, guided by explicit evaluative standards, and characterized by substantial outcome uncertainty. Such features are often accompanied by heightened attention to self-performance and increased sensitivity to potential shortcomings. This pattern aligns closely with the evaluative sensitivity and error focused tendencies observed among individuals with higher levels of negative perfectionism. At the same time, the study by Zhang et al. (2025) shows that perfectionism characteristics related to stronger self-management and goal maintenance are associated with college students maintaining motivation and a sense of direction when facing challenges, and they are also accompanied by lower stress experiences in some situations. Besides, Kim et al. (2022) observe that in highly evaluative settings such as job related examinations and interviews, high standard self-monitoring may also coincide with increased attention to performance maintenance, which is often accompanied by greater psychological burden. Taken together, different dimensions of perfectionism may relate to employment stress through complex emotional and cognitive patterns in job search contexts. Examining the relationships between different dimensions of perfectionism and employment stress among college students helps to deepen the understanding of how perfectionism is associated with employment stress. It also has important research significance.

#### 2.2.2 The mediating role of career adaptability

Career adaptability reflects individuals’ level of readiness and coping ability when they face career development tasks, career role transitions, and uncertain career situations (Savickas, 1997). From this perspective, career adaptability is not only a general positive trait, but also a key psychological resource that is closely related to the process of career transition. Existing research suggests that career adaptability relates to employment related stress through both behavioral and emotional pathways. In a three-wave longitudinal study of graduating college students, Ouyang et al. (2025) reported that higher levels of career adaptability at the initial stage were associated with more active job search behaviors at later stages and with higher subjective wellbeing. Although this study did not directly assess employment stress, the findings suggest that career adaptability may relate to job search experiences through enhanced agency and perceived control, which often accompany lower levels of tension during the job search process. Evidence from work contexts offers further support for the stress related relevance of career adaptability. For example, Marcionetti et al. (2025) found a significant association between career adaptability and lower levels of perceived work stress. Individuals with higher career adaptability more often appraised work situations as manageable rather than stressful. Taken together, these studies indicate that career adaptability is associated with stress experiences through multiple pathways. It commonly co-occurs with more active job search engagement and with reduced threat perceptions linked to career uncertainty, both of which are accompanied by greater emotional stability.

Career adaptability is not a fixed and stable trait, but a dynamic capacity that develops through ongoing interactions between the individual and the environment. Savickas (1997) suggested that career adaptability is gradually formed as individuals cope with both predictable career tasks and unpredictable adjustment demand. Therefore, it may be strengthened through effective coping, but also weakened by prolonged adversity or insufficient support. Accordingly, different perfectionistic tendencies may show different associations with career adaptability. Chen et al. (2022) reported that positive perfectionism shows a significant association with higher levels of career adaptability among college students, whereas negative perfectionism displays an opposite pattern of association. Individuals with a stronger tendency toward orderliness and structure are often more likely to maintain a relatively good level of psychological resources in career planning, goal adjustment, exploration, and confidence. In contrast, individuals who focus excessively on others’ evaluations or experience strong external expectations more often report signs of depletion in career adaptability resources. This pattern aligns with findings by Tang et al. (2026). Positive perfectionism is more likely to be associated with a better career readiness state, clearer sense of direction, and stronger ability to mobilize resources. Negative perfectionism is more likely to be associated with adaptation stress when individuals deal with career tasks, uncertainty, and career development choices. Taken together, these findings suggest that perfectionism may accompany both resource facilitation and resource depletion during career development. Although direct evidence remains limited regarding the mediating role of career adaptability between perfectionism and employment stress, existing research indicates that perfectionism is closely associated with how individuals mobilize and allocate resources in career development contexts. These resource related patterns may, in turn, show indirect associations with stress experiences during the job search stage, providing a theoretical basis for the hypotheses proposed in the present study.

## 3 The present study

**Figure 1:**
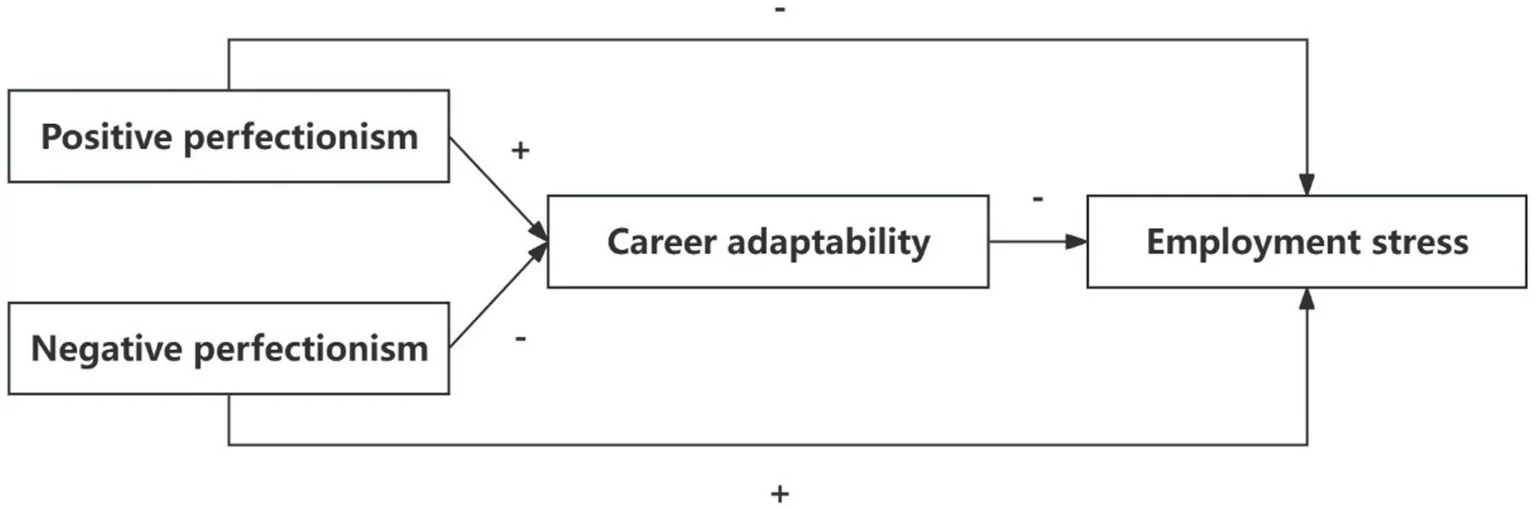
Hypothetical model. The “+” and “−” signs in the figure indicate the expected direction of the hypothesized paths, where “+” denotes a positive relationship and “−” denotes a negative relationship, rather than specific effect sizes.

Building on prior research, the present study adopts COR theory as its theoretical framework to examine associations between different dimensions of perfectionism and employment stress among college students. The study further incorporates career adaptability into the model as a potential mediating mechanism. Based on this framework, a conceptual model was developed (Figure 1), and the following hypotheses were subjected to empirical examination.

H1: Positive perfectionism is significantly negatively associated with employment stress among college students.

H2: Negative perfectionism is significantly positively associated with employment stress among college students.

H3: Career adaptability shows a mediating role in the association between positive perfectionism and employment stress among college students.

H4: Career adaptability shows a mediating role in the association between negative perfectionism and employment stress among college students.

## 4 Method

### 4.1 Sample and data collection

The study was conducted between February and March in 2026 using an anonymous online survey via the questionnaire platform.^1^ Following the sample size estimation guideline proposed by Kline (2018), which recommends a minimum of 10 participants per item with an additional allowance of approximately 20 percent, the target sample size was set at 828 college students. Participants were recruited from three comprehensive universities in Yunnan and Guizhou, western China. During the sampling process, participants were randomly selected from the student rosters provided by each cooperating university. Due to differences in roster structures and classification criteria across universities, definitions of academic year and major were not fully consistent; therefore, a unified stratification method was not employed. It should be noted that the sample of this study was drawn from a limited number of universities within a specific region, and does not constitute a probability sample based on a broader population framework. Caution is warranted when generalizing the findings to other universities or regions. Then, counselors or course instructors from each university distributed the study information through class groups, grade groups, or internal campus communication platforms, and sent questionnaire links or QR codes to the selected students. Because this study included participants under the age of 18, written informed consent was obtained in advance from their legal guardians or close relatives. At the same time, written study information and informed consent were provided on the first page of the questionnaire. Participants could only proceed to the formal survey after giving clear consent. They could also withdraw at any time during completion without any negative consequences. The whole research process followed relevant ethical guidelines and strictly protected the confidentiality and privacy of participants.

A total of 965 questionnaires were distributed in this study, and 912 were returned, with a response rate of 94.5%. During the data cleaning stage, the research team followed the data quality control recommendations proposed by Boateng et al. (2018) to screen the returned questionnaires. Questionnaires with more than 50% missing responses on key items were excluded (*n* = 4). Questionnaires showing abnormal response patterns were also excluded (*n* = 8). Abnormal response patterns were defined as continuous selection of the same option longer than half of the total number of items in a scale. In addition, although questionnaire instructions recorded completion time as a general behavioral indicator, completion time was not used as a strict exclusion criterion. This decision was made because response time may vary substantially across individuals due to differences in reading speed, cognitive processing, and response strategies, and a single threshold may lead to the unnecessary exclusion of valid responses. Therefore, data quality control primarily relied on missing data and response pattern detection rather than time-based screening. After these screening procedures, 900 valid questionnaires were retained, meeting the sample requirements for subsequent analyses. The relatively high validity rate may be related to the sample group and survey context. The participants were college students, who generally have relatively strong comprehension ability, and the survey topic was closely related to their academic development and job preparation. Therefore, participants usually showed higher engagement in responding. At the same time, the questionnaires were distributed through relatively centralized on-campus organizational channels, which also to some extent reduced invalid responses. Descriptive statistics for the sample appear in Table 1. The sample shows relatively balanced distributions across gender, age, place of origin, and academic discipline. Further group comparisons indicate that employment stress does not differ significantly across these demographic characteristics. It should be noted that, due to the anonymous online survey design and the absence of a school identifier variable, the specific sample composition across universities could not be distinguished at the data level. Given this data structure, data from multiple universities were combined and analyzed as a single pooled sample, focusing on individual-level psychological mechanisms rather than institutional-level comparisons or cross-university differences.

**Table 1:** Demographic characteristics of the sample.

Demographic characteristic	Category	Quantity	Percentage	Mean	Standard deviation	ES (*p*)
Gender	Male	404	44.9%	2.947	0.546	0.657
	Female	496	55.1%	2.931	0.542	
Age	17–18	86	9.6%	2.976	0.551	0.692
	19–20	315	35.0%	2.952	0.558	
	21–22	283	31.4%	2.909	0.542	
	≥23	216	24.0%	2.940	0.522	
Hometown	Rural	327	36.3%	2.936	0.533	0.938
	Urban	573	63.7%	2.940	0.550	
Academic disciplines	Humanities and Social Sciences	270	30.0%	2.967	0.575	0.507
	Natural Sciences	295	32.8%	2.911	0.524	
	Engineering and Technology	186	20.7%	2.962	0.510	
	Arts	149	16.6%	2.908	0.563	

ES: employment stress.

### 4.2 Measures

All instruments used in the present study are well established scales, and the original item content and scale structure were retained. All measures employed a five-point Likert response format, with higher scores indicating higher levels of the assessed constructs.

The study used the Frost Multidimensional Perfectionism Scale developed by Frost et al. (1990), using its Chinese version that has been translated and revised (Chen et al., 2022; Cheng et al., 1999). The scale includes 27 items across five dimensions: concern over mistakes, personal standards, parental expectations, doubts about action, and organization. It should be noted that perfectionism is a multidimensional construct. Prior studies have proposed different classical dimensional models and have also offered different interpretations of the higher-order grouping of subscales in the Frost Multidimensional Perfectionism Scale (Hewitt and Flett, 1991; Stoeber, 2018). Some studies on perfectionism often consider personal standards as a component of perfectionistic strivings, while concern over mistakes and doubts about actions are regarded as key components of perfectionistic concerns (Stoeber, 2018). At the same time, Frost et al. (1990) pointed out that organization mainly reflects individuals’ emphasis on orderliness and structure, and this component is usually associated with fewer maladaptive characteristics. Given that the Chinese version used in this study and prior related studies adopted a scoring approach in which concern over mistakes, personal standards, parental expectations, and doubts about actions were combined as negative perfectionism, while organization was classified as positive perfectionism (Chen et al., 2022; Cheng et al., 1999; Tang et al., 2026), this study follows this operationalization. In terms of theoretical meaning, this classification is not intended to provide a general definition of the higher-order structure of perfectionism. Instead, it aims to distinguish between a set of adaptive components that emphasize orderliness and structured tendencies, and a set of non-adaptive components that are more likely to be associated with concern over mistakes, external expectations, doubts about actions, and overly high self-demands. In this study, negative perfectionism includes 21 items. A representative item is “I set higher goals than most people around me.” Positive perfectionism consists of 6 items from the organization dimension. A representative item is “I try to be an organized person.” In the present study, Cronbach’s *α* values were 0.956 for negative perfectionism and 0.855 for positive perfectionism.

Career adaptability was assessed using the Career Adapt-Abilities Scale developed by Savickas and Porfeli (2012) and validated among Chinese college students by Hou et al. (2012). The scale consists of 24 items measuring four dimensions: career concern, career control, career curiosity, and career confidence, with six items for each dimension. A representative item is “I care about my career development.” In the current sample, Cronbach’s *α* values were 0.866, 0.872, 0.869, and 0.876 for the four dimensions, respectively, and 0.964 for the total scale.

Employment stress was measured using the Employment Stress Scale developed and adapted for Chinese college students by Liu (2010). The scale includes 14 items across four dimensions: family factors with four items, school factors with four items, major related factors with three items, and self-related factors with three items. A representative item is “My language expression ability is not good enough.” This scale has been widely used in China (Wu et al., 2024; Yang et al., 2022). In the present study, Cronbach’s *α* values were 0.827, 0.828, 0.756, and 0.781 for the four subscales, respectively, and 0.943 for the total scale.

### 4.3 Data analysis

Given that the model included 69 measurement items, Smart PLS provides an appropriate analytical approach for handling multi-indicator constructs, evaluating the reliability and validity of the measurement model, and performing variance based structural equation modeling. Accordingly, the study used Smart PLS to assess both the measurement model and the structural model. During the structural model evaluation process, to improve the stability of data analysis and the accuracy of effect testing, this study used the bootstrap resampling method for estimation. A total of 5,000 resamples were conducted to test the structural paths and indirect effects.

## 5 Results

### 5.1 Descriptive statistics of variables

Table 2 presents the descriptive statistics, correlation coefficients, and internal consistency of the main variables. The results show that positive perfectionism is significantly positively associated with career adaptability and is significantly negatively associated with employment stress. Negative perfectionism is significantly negatively associated with career adaptability and is significantly positively associated with employment stress. Career adaptability is significantly negatively associated with employment stress. It is worth noting that there is no significant correlation between positive perfectionism and negative perfectionism, which indicates a good level of discriminant validity in this study. The Cronbach’s alpha coefficients of all variables are above 0.85, indicating good internal consistency of the scales (Hair et al., 2021). It should be noted that some Cronbach’s alpha values in Table 2 are relatively high. Considering that Cronbach’s alpha may be affected by the number of items and the similarity of item content, this study will further use composite reliability (CR), average variance extracted (AVE), heterotrait–monotrait ratio (HTMT), Fornell–Larcker criterion, and variance inflation factor (VIF) to comprehensively test internal consistency, convergent validity, discriminant validity, and multicollinearity.

**Table 2:** Descriptive statistics, correlation coefficients, and internal consistency of main variables.

Variables	M ± SD	PP	NP	CA	ES	Cronbach’s *α*
PP	2.993 ± 0.529	1				0.855
NP	3.038 ± 0.519	−0.016	1			0.956
CA	3.087 ± 0.516	0.429***	−0.336***	1		0.964
ES	2.938 ± 0.543	−0.366***	0.437***	−0.618***	1	0.943

***p < 0.001, PP, positive perfectionism; NP, negative perfectionism; CA, career adaptability; ES, employment stress.

### 5.2 Measurement model

Detailed results of the reliability and validity analyses of the scales are provided in the Supplementary Materials. All item loadings exceed 0.708, and all constructs show CR values above 0.700. The AVE values for all constructs exceed 0.50. In addition, all HTMT values remain below 0.85, and the square roots of the AVE values for each construct exceed the corresponding inter construct correlation coefficients. Together, these results indicate satisfactory reliability and validity for the measurement model (Hair et al., 2021).

### 5.3 Confirmatory factor analysis

The results of the confirmatory factor analysis indicate a good overall model fit. Specifically, the *χ*^2^ to df ratio = 1.020 and remains below the recommended threshold of 2. The RMSEA = 0.005 and falls below 0.08. In addition, GFI = 0.936, NFI = 0.943, CFI = 0.998, IFI = 0.998, and TLI = 0.998, with all indices exceeding the recommended cutoff of 0.900 (Goretzko et al., 2024).

### 5.4 Common method Bias

To test common method bias, this study used the common latent factor method within a confirmatory factor analysis framework. Specifically, a common method latent factor was added to the original measurement model, and its variance was fixed at 1. Then, two nested models were constructed. In Model 1, the paths from the common method factor to each observed item were fixed at 0. In Model 2, these paths were constrained to be equal and estimated as a single parameter. The chi-square difference between the two models was used to assess common method bias. The results showed that the difference between the models was not statistically significant (Δ*χ*^2^ = 3.303, Δdf = 1, *p* > 0.05). These findings suggest that common method bias is unlikely to represent a substantial concern in the present study (Williams et al., 2010).

### 5.5 Structural model

Detailed results of the multicollinearity assessment are provided in the Supplementary Materials. The maximum VIF equals 1.427 and remains below the threshold of 3, indicating that multicollinearity does not pose a concern in the structural model (Hair and Alamer, 2022).

Table 3 presents the results of the path analysis. The results show that negative perfectionism is significantly negatively associated with career adaptability and significantly positively associated with employment stress. Positive perfectionism shows a significant negative association with employment stress and a significant positive association with career adaptability. In addition, career adaptability is significantly negatively associated with employment stress.

**Table 3:** Paths of the structural model.

Hypothesis	*β*	*T*	*p*	95% confidence interval		Results
				Lower bound	Upper bound	
CA → ES	−0.450	17.862	<0.001	−0.499	−0.400	Supported
NP → CA	−0.331	13.026	<0.001	−0.383	−0.284	Supported
NP → ES	0.286	10.789	<0.001	0.234	0.338	Supported
PP → CA	0.424	16.827	<0.001	0.374	0.472	Supported
PP → ES	−0.168	6.701	<0.001	−0.217	−0.119	Supported

PP, positive perfectionism; NP, negative perfectionism; CA, career adaptability; ES, employment stress.

The *R*^2^ results show that the model explains 29.5% of the variance in career adaptability (*R*^2^ = 0.295, adjusted R^2^ = 0.293) and 46.8% of the variance in employment stress (*R*^2^ = 0.468, adjusted *R*^2^ = 0.463). In addition, the *Q*^2^ values of career adaptability and employment stress are 0.160 and 0.265, respectively, both above 0, indicating that the model has acceptable explanatory power and predictive relevance (Hair et al., 2021).

After accounting for latent constructs, career adaptability shows a mediating role in the associations between negative perfectionism and employment stress, as well as between positive perfectionism and employment stress (Table 4).

**Table 4:** Mediation analysis of career adaptability.

Path	*β*	*T*	*p*	95% confidence interval	
				Lower bound	Upper bound
PP → ES					
Total effects	−0.359	14.536	<0.001	−0.406	−0.310
Direct effects	−0.168	6.701	<0.001	−0.217	−0.119
Indirect effects	−0.191	11.722	<0.001	−0.224	−0.159
NP → ES					
Total effects	0.435	16.918	<0.001	0.385	0.487
Direct effects	0.286	10.789	<0.001	0.234	0.338
Indirect effects	0.149	10.894	<0.001	0.124	0.178

PP, positive perfectionism; NP, negative perfectionism; ES, employment stress.

Although the present study found no significant association between gender and employment stress, this does not imply the absence of significant differences in path relationships across gender groups within the model. Therefore, this study conducted a multi-group analysis of gender to examine whether the path relationships between dimensions of perfectionism and employment stress differ across gender groups. Prior to performing the multi-group analysis, the measurement invariance of the measurement model across groups was tested using the MICOM procedure (Henseler et al., 2016). Step 1 was supported by default. The results of Step 2 showed that career adaptability (*p* = 0.531), employment stress (*p* = 0.741), and positive perfectionism (*p* = 0.927) passed the permutation test (*p* > 0.05), whereas negative perfectionism (*p* = 0.021) did not pass the compositional invariance test. Steps 3a and 3b indicated that both mean and variance invariance were established for all constructs (*p* > 0.05).

The multi-group analysis results (Table 5) indicated that the coefficients of all direct and indirect paths in the model did not differ significantly between male and female students. Specifically, the associations of positive perfectionism and negative perfectionism with career adaptability and employment stress, as well as the indirect associations through career adaptability, demonstrated homogeneity across gender groups. No significant gender differences were identified for paths involving constructs that established compositional invariance. Results involving negative perfectionism should be interpreted cautiously because compositional invariance was not established for this construct.

**Table 5:** Multigroup path analysis (male and female).

Path	Male	Female	Original difference	Mean difference of permutation	5.0%	95.0%
CA → ES	−0.436	−0.461	0.025	−0.003	−0.101	0.098
NP → CA	−0.317	−0.347	0.030	−0.002	−0.102	0.101
NP → ES	0.27	0.296	−0.024	−0.001	−0.106	0.101
PP → CA	0.447	0.408	0.038	0.001	−0.106	0.103
PP → ES	−0.212	−0.132	−0.080	0.001	−0.099	0.100
NP → CA → ES	0.138	0.160	−0.022	0.002	−0.053	0.055
PP → CA → ES	−0.195	−0.188	−0.006	−0.002	−0.069	0.069

PP, positive perfectionism; NP, negative perfectionism; CA, career adaptability; ES, employment stress.

To examine the robustness of the model’s directional assumptions, a reverse mediation model was further specified, in which the original pathways were reversed (i.e., employment stress as the independent variable, career adaptability as the mediator, and perfectionism as the dependent variable). The same bootstrap procedure (5,000 resamples) was applied for estimation. The results are presented in Table 6. Regarding overall model fit, the SRMR values for both models were below 0.08, indicating acceptable overall fit; the theoretical model (SRMR = 0.027) showed a better fit than the reverse model (SRMR = 0.035). In terms of the comparison of common paths, the path coefficients from positive perfectionism to career adaptability (*β* = 0.424) and from negative perfectionism to career adaptability (*β* = −0.331) in the theoretical model were both stronger than those in the reverse model (*β* = 0.329 and −0.107, respectively), suggesting that the effects of perfectionism on career adaptability were more stable in the theoretical model. It should be noted that although the *R*^2^ and *Q*^2^ values of the theoretical model were generally better than those of the reverse model, the two models involved different outcome variables; therefore, these values are provided for reference only and should not be taken as strong evidence. Meanwhile, all the above comparisons can only offer supplementary support for the proposed theoretical specification and cannot establish temporal or causal order among the variables (Hair et al., 2021; Hair et al., 2019; Shmueli et al., 2019).

**Table 6:** Comparison of the theoretical model and the reverse mediation model.

Indicator	Theoretical model	Reverse model
*β* (PP → CA)	0.424	0.329
*β* (NP → CA)	−0.331	−0.107
*β* (PP → ES)	−0.168	−0.163
*β* (NP → ES)	0.286	0.374
*β* (CA → ES)	−0.450	−0.620
*R*^2^ (outcome variable)	0.468	0.201 (PP)
		0.201 (NP)
*R*^2^ (mediator variable)	0.295	0.384
SRMR	0.027	0.035
*Q*^2^ (outcome variable)	0.265	0.115 (PP)
		0.105 (NP)
*Q*^2^ (mediator variable)	0.160	0.209

PP, positive perfectionism; NP, negative perfectionism; CA, career adaptability; ES, employment stress.

## 6 Discussion

The results of the present study show that positive perfectionism is significantly negatively associated with employment stress among college students, whereas negative perfectionism is significantly positively associated with employment stress, supporting H1 and H2. Specifically, college students with higher levels of positive perfectionism tend to report lower levels of employment stress, whereas those with higher levels of negative perfectionism more often report elevated stress experiences. These patterns align with findings reported in prior research (Kwak et al., 2025; Powell et al., 2021; Zhang et al., 2025). From the perspective of COR theory, these results can be understood as reflecting differences in how distinct perfectionism orientations relate to patterns of psychological resource investment and perceived resource consumption, which co-occur with variations in employment stress experiences. Job search contexts typically involve substantial resource demands, as college students need to continuously mobilize emotional, cognitive, and behavioral resources during preparation and decision making. Positive perfectionism is associated with a relatively organized and stable way of investing resources, which is associated with reduced inefficient resource loss and is more likely to be associated with lower employment stress (Zhang et al., 2025). In contrast, negative perfectionism is more likely to be associated with excessive resource consumption in highly competitive situations, and it is more likely to be associated with higher levels of stress experience (Kwak et al., 2025; Powell et al., 2021). Considering the current context faced by college students, intensified labor market competition and increased sensitivity to external evaluation may further accentuate stress related differences associated with distinct perfectionism orientations. College students with higher levels of positive perfectionism often maintain relatively stable psychological states when confronted with uncertainty in recruitment information and external feedback, and their perceived employment stress remains more manageable. By contrast, students with higher levels of negative perfectionism are more susceptible to others’ evaluations and outcome comparisons. During the job search process, they frequently monitor whether their performance meets expected standards, which is often accompanied by more frequent experiences of tension and anxiety related to employment stress. Compared with previous research that mainly focused on general stress or academic stress, this study further suggests that the association between perfectionism and stress experience also appears in the more situational context of job transition. Positive perfectionism and negative perfectionism show opposite directions of association with employment stress. This finding suggests that perfectionism cannot be simply viewed as a single risk or protective factor. Different dimensions of perfectionism may be associated with different meanings of psychological adjustment in the job transition stage. At the same time, this also provides practical implications for university career guidance and psychological support. When helping college students cope with employment stress, attention should be paid to different perfectionism tendencies, and more targeted support strategies should be used.

The findings of the present study indicate that career adaptability shows a mediating role in the associations between different dimensions of perfectionism and employment stress among college students, supporting H3 and H4. Specifically, college students with higher levels of positive perfectionism usually show higher career adaptability and are also associated with lower employment stress. In contrast, those with higher levels of negative perfectionism are more likely to show lower career adaptability and are also associated with higher employment stress. This result is generally consistent with previous studies (Chen et al., 2022; Marcionetti et al., 2025). As students enter the job search stage, they continuously process recruitment information, prepare application materials, and respond to selection outcomes, while also balancing academic demands, interpersonal relationships, and family expectations. In this context, college students with a higher level of positive perfectionism are more likely to maintain a relatively stable preparation pace. They also tend to have a stronger sense of direction and coping when facing complex tasks (Chen et al., 2022). Therefore, they may be associated with higher career adaptability and lower employment stress. In contrast, individuals with higher levels of negative perfectionism are more likely to repeatedly focus on whether they have done well enough and whether they have met external expectations during the job search process. This is associated with hesitation or repeated adjustment in preparation and action (Suh and Flores, 2023). For college students, this not only may disrupt their original preparation rhythm, but it may also weaken their sense of control over career tasks, and it may be associated with higher levels of tension, anxiety, and stress experience. The results of this study suggest that career adaptability may be an important correlate in examining the relationship between personality tendencies and employment stress experience. This indicates that, in the employment context, career adaptability may help to better understand how different perfectionism tendencies are associated with employment stress. Accordingly, in university career guidance and psychological intervention, career adaptability can be considered an important aspect of attention, which may provide reference for psychological adjustment and developmental support during the job transition stage of college students.

The findings of this study also need to be understood in relation to specific cultural and institutional contexts. In the Chinese context, the employment transition of college students is not only related to the development of personal interests and abilities, but is also influenced by family expectations, social evaluation, and the competitiveness of the employment environment (Wu et al., 2024; Yang et al., 2022). In a cultural context that places strong emphasis on social recognition and “face” value, employment outcomes are more likely to be assigned meanings beyond individual development, such as family reputation and social comparison. This makes college students more likely to closely link self-worth with external standards, others’ evaluations, and future outcomes. At the same time, in the Chinese employment context, educational background, school level, exam performance, and the possibility of entering relatively stable job positions often have high importance (Yu et al., 2023). This may also lead college students to pay more attention to performance outcomes and their social meaning during the job search process. It should be noted that the sample of this study was drawn solely from three universities in Yunnan and Guizhou provinces in western China, with a relatively concentrated geographical scope. This, to some extent, limits the generalizability of the findings to other regions, such as those in the eastern coastal areas or first-tier cities. Since this study did not differentiate the specific sample composition across universities at the data level, it was not possible to include university fixed effects or conduct stratified analyses in the model. This limitation may affect the precise characterization of institution-level differences. Given the cultural and institutional context described above, the relationships among the variables examined in this study may, to some extent, reflect culturally embedded psychological mechanisms characteristic of the employment transition process among college students in Yunnan and Guizhou within the Chinese context. At the same time, these methodological limitations suggest that the interpretation of the relevant results should be approached with caution and situated within an individual-level analytical framework. Future research could incorporate multi-level data or stratified sampling designs, along with institution-level variables or multilevel modeling, across different cultural, regional, and institutional contexts, to further examine the cross-contextual stability and explanatory boundaries of the above relationships.

Finally, this study also found that the path relationships in the model did not differ significantly across gender groups. This may be because the present study focused on employment stress as a specific situational context rather than on generalized emotional distress or mental health indicators, and such context specificity may have attenuated the manifestation of gender differences (Gao et al., 2020). In the Chinese college student employment market, male and female students face relatively homogeneous competitive pressures and evaluative standards, and the consistency of external stressors may have masked the gender differentiation in internal trait responses (Peng et al., 2024). In addition, the sample of this study was drawn from three comprehensive universities in Yunnan and Guizhou provinces in western China, and the shared sociocultural context may have further reduced heterogeneity across gender groups.

## 7 Implications and limitations

### 7.1 Implications

Grounded in COR theory, the present study advances understanding of stress experiences during the job search stage among college students from a resource-based perspective. First, the study extends the application of COR theory beyond traditional work settings to the stages of career preparation and transition. The findings indicate that college students also experience patterns of psychological resource accumulation, depletion, and reallocation while engaging in job search tasks. This extension offers empirical support for applying the theory within educational and career development contexts. Second, the study highlights the role of perfectionism orientations in resource related experiences. It illustrates how individual differences in personality traits relate to variations in how students perceive and experience resources in employment contexts. In doing so, the findings enrich theoretical discussions within COR theory regarding individual variability in resource acquisition and resource constraint patterns. In addition, by incorporating career adaptability into the analytical framework as a process oriented psychological construct that reflects resource reserve status, the study elaborates the resource linkage between trait level tendencies and situational stress experiences. This approach presents a more detailed depiction of resource circulation processes at the level of psychological mechanisms. Overall, within the existing theoretical framework, the present study adds evidence supporting the relevance of COR theory during the job search stage among college students and offers useful reference points for examining career development and psychological adjustment from a resource-based perspective.

The findings of this study may provide some reference for supporting college students during the job search stage. For universities, career-related courses may pay attention to students’ perfectionism tendencies, help them identify their behavioral patterns and psychological experiences in job preparation, and provide more targeted guidance accordingly. For example, students with a stronger tendency toward organized preparation and stable progress may be further supported in maintaining clear goals and a stable preparation pace. Students who are more likely to experience repeated worry, hesitation, or excessive focus on external evaluation may be supported through task breakdown, reduction of repeated revision, and situational practice. For career guidance centers, career adaptability can be considered an important focus. Through activities such as simulated interviews, career exploration, and short-term planning, students may be helped to strengthen their sense of readiness, direction, and coping in job search tasks (Kim, 2022). For counselors and mental health practitioners, the association patterns presented in this study may serve as a reference for identifying students at higher risk of stress. For students who continuously show repeated hesitation, excessive worry, or difficulty in task progress in job search-related issues, stress management, emotional regulation, or supportive counseling may be considered (Lee and Jung, 2022). From the family perspective, reducing unnecessary comparisons, providing appropriate encouragement, and assisting in information screening may help create a more stable supportive environment for job seeking (Wu et al., 2024). Overall, the findings of this study may provide reference for understanding the psychological characteristics and related support needs of college students during the job search stage, and may also offer some implications for future practical work.

### 7.2 Limitations and future research directions

Although the present study provides new evidence regarding associations among different dimensions of perfectionism, career adaptability, and employment stress among college students, several limitations warrant consideration. First, this study used a cross-sectional design, which is suitable for presenting associations between variables, but it cannot support strict causal inferences. Future studies may use longitudinal or multiple-wave designs and combine actual job search outcomes or employment-related behaviors to further examine the relationships among these variables. Second, following the approach adopted in some previous studies, this study classified perfectionism into positive and negative types, and assigned personal standards to the negative perfectionism dimension. However, this classification may not fully capture the richness of the perfectionism construct. Future research could draw upon more established theoretical perspectives and adopt more fine-grained distinctions to further explore the roles of different perfectionism components. Third, the sample of this study was drawn exclusively from college students in Yunnan and Guizhou provinces in western China; therefore, the findings may be influenced by the specific cultural and institutional context of these regions. For example, cultural factors such as family expectations and social evaluation, as well as the emphasis on stable job positions, may play a role. Therefore, the generalizability of the findings should be interpreted with caution. Future studies may conduct cross-cultural replication in different cultural and educational contexts to examine the stability of the findings. Fourth, employment stress was assessed using a scale originally developed in an unpublished master’s thesis. Although the scale has subsequently been used in studies involving Chinese college students and demonstrated satisfactory internal consistency in the present sample, its original source and limited international validation may restrict the comparability of the findings across cultural and educational contexts. Future research could replicate the present findings using internationally established employment-related stress measures or further examine the psychometric properties of this scale across diverse student populations. Finally, this study did not further consider factors such as university type, region, graduation year, current job search status, preparation for graduate study or civil service exams, and family economic status. These factors may be associated with college students’ employment stress, career adaptability, and employment resources to some extent. Therefore, future research may include these variables for a more fine-grained analysis.

## 8 Conclusion

Grounded in COR theory, the present study examined the association structure among different orientations of perfectionism, career adaptability, and employment stress among college students. The results show that positive perfectionism displays a consistent association with lower levels of employment stress, whereas negative perfectionism more often co-occurs with higher levels of employment stress. In addition, career adaptability demonstrates a mediating role in the associations between both forms of perfectionism and employment stress. These findings suggest that distinct perfectionism orientations correspond to different patterns of resource experiences during the job search stage, and that these patterns relate to students’ stress experiences through career adaptability as a process oriented psychological resource. Overall, the present study enhances understanding of variability in resource experiences among college students during the job search stage and offers useful reference points for interpreting employment stress from a resource-based perspective.

## Data Availability

The raw data supporting the conclusions of this article will be made available by the authors, without undue reservation.

## Footnotes

1
www.sojump.com
