# Supplementary material for: The double-edged nature of multidimensional perfectionism: career adaptability in employment stress among college students
Source: Front Psychol. 2026 Aug 31;17:1845501. doi: 10.3389/fpsyg.2026.1845501 (PMC13572112; doi:10.3389/fpsyg.2026.1845501)
Supplement: Supplementary file 1 [file Table_1.DOCX]

Table S1 Reliability and Validity

| Constructs | Items | Loadings | CR | Cronbach’s α | AVE |
| --- | --- | --- | --- | --- | --- |
| PP | PP 1 | 0.780 | 0.892 | 0.855 | 0.579 |
|  | PP 2 | 0.758 |  |  |  |
|  | PP 3 | 0.764 |  |  |  |
|  | PP 4 | 0.774 |  |  |  |
|  | PP 5 | 0.737 |  |  |  |
|  | PP 6 | 0.753 |  |  |  |
| NP | NP 1 | 0.711 | 0.959 | 0.956 | 0.530 |
|  | NP 2 | 0.725 |  |  |  |
|  | NP 3 | 0.735 |  |  |  |
|  | NP 4 | 0.756 |  |  |  |
|  | NP 5 | 0.744 |  |  |  |
|  | NP 6 | 0.720 |  |  |  |
|  | NP 7 | 0.725 |  |  |  |
|  | NP 8 | 0.713 |  |  |  |
|  | NP 9 | 0.733 |  |  |  |
|  | NP 10 | 0.723 |  |  |  |
|  | NP 11 | 0.736 |  |  |  |
|  | NP 12 | 0.741 |  |  |  |
|  | NP 13 | 0.724 |  |  |  |
|  | NP 14 | 0.712 |  |  |  |
|  | NP 15 | 0.722 |  |  |  |
|  | NP 16 | 0.711 |  |  |  |
|  | NP 17 | 0.736 |  |  |  |
|  | NP 18 | 0.729 |  |  |  |
|  | NP 19 | 0.735 |  |  |  |
|  | NP 20 | 0.728 |  |  |  |
|  | NP 21 | 0.722 |  |  |  |
| CA | CA 1 | 0.745 | 0.967 | 0.964 | 0.551 |
|  | CA 2 | 0.749 |  |  |  |
|  | CA 3 | 0.744 |  |  |  |
|  | CA 4 | 0.722 |  |  |  |
|  | CA 5 | 0.739 |  |  |  |
|  | CA 6 | 0.716 |  |  |  |
|  | CA 7 | 0.742 |  |  |  |
|  | CA 8 | 0.729 |  |  |  |
|  | CA 9 | 0.735 |  |  |  |
|  | CA 10 | 0.742 |  |  |  |
|  | CA 11 | 0.749 |  |  |  |
|  | CA 12 | 0.733 |  |  |  |
|  | CA 13 | 0.754 |  |  |  |
|  | CA 14 | 0.735 |  |  |  |
|  | CA 15 | 0.725 |  |  |  |
|  | CA 16 | 0.741 |  |  |  |
|  | CA 17 | 0.738 |  |  |  |
|  | CA 18 | 0.744 |  |  |  |
|  | CA 19 | 0.751 |  |  |  |
|  | CA 20 | 0.752 |  |  |  |
|  | CA 21 | 0.741 |  |  |  |
|  | CA 22 | 0.742 |  |  |  |
|  | CA 23 | 0.738 |  |  |  |
|  | CA 24 | 0.761 |  |  |  |
| ES | ES 1 | 0.734 | 0.949 | 0.943 | 0.573 |
|  | ES 2 | 0.747 |  |  |  |
|  | ES 3 | 0.757 |  |  |  |
|  | ES 4 | 0.746 |  |  |  |
|  | ES 5 | 0.753 |  |  |  |
|  | ES 6 | 0.775 |  |  |  |
|  | ES 7 | 0.745 |  |  |  |
|  | ES 8 | 0.777 |  |  |  |
|  | ES 9 | 0.794 |  |  |  |
|  | ES 10 | 0.775 |  |  |  |
|  | ES 11 | 0.756 |  |  |  |
|  | ES 12 | 0.738 |  |  |  |
|  | ES 13 | 0.750 |  |  |  |
|  | ES 14 | 0.748 |  |  |  |

Note: PP Positive perfectionism, NP Negative perfectionism, CA Career adaptability, ES Employment stress

Table S2 Discriminant validity (HTMT criterion)

|  | CA | ES | NP | PP |
| --- | --- | --- | --- | --- |
| CA |  |  |  |  |
| ES | 0.649 |  |  |  |
| NP | 0.350 | 0.461 |  |  |
| PP | 0.473 | 0.409 | 0.044 |  |

Note: PP Positive perfectionism, NP Negative perfectionism, CA Career adaptability, ES Employment stress

Table S3 Discriminant validity (Fornell–Larcker criterion)

|  | CA | ES | NP | PP |
| --- | --- | --- | --- | --- |
| CA | ***0.742*** |  |  |  |
| ES | -0.620 | ***0.757*** |  |  |
| NP | -0.339 | 0.440 | ***0.728*** |  |
| PP | 0.430 | -0.367 | -0.018 | ***0.761*** |

Note: The square roots of the AVE values are presented as the bold italicized diagonal elements, PP Positive perfectionism, NP Negative perfectionism, CA Career adaptability, ES Employment stress

Table S4 VIF

|  | CA | ES | NP | PP |
| --- | --- | --- | --- | --- |
| CA |  | 1.427 |  |  |
| ES |  |  |  |  |
| NP | 1.000 | 1.169 |  |  |
| PP | 1.000 | 1.258 |  |  |

Note: PP Positive perfectionism, NP Negative perfectionism, CA Career adaptability, ES Employment stress
